# Preoperative anxiety during COVID-19 pandemic: A single-center observational study and comparison with a historical cohort

**DOI:** 10.3389/fmed.2022.1062381

**Published:** 2022-12-15

**Authors:** Pasquale Buonanno, Annachiara Marra, Carmine Iacovazzo, Maria Vargas, Serena Nappi, Andrea Uriel de Siena, Giuseppe Servillo

**Affiliations:** Department of Neurosciences, Reproductive and Odontostomatological Sciences, University of Naples “Federico II”, Naples, Italy

**Keywords:** anxiety, preoperative period, test anxiety scale, psychology, COVID-19 pandemic

## Abstract

**Background:**

Preoperative anxiety is a common sensation experienced by patients undergoing surgical interventions. It can influence intraoperative and postoperative management through the activation of the neuroendocrine system, leading to tachycardia, hypertension, pulmonary complications, higher consumption of anesthetic drugs, and increased postoperative pain. Our aim was to investigate the level of preoperative anxiety during the COVID-19 pandemic; we also compared it to the preoperative anxiety of a historical cohort before the outbreak.

**Methods:**

This is a single-center observational study. We enrolled 314 patients during the pandemic from May 2021 to November 2021, and our historical cohort consisted of 122 patients enrolled from July 2015 to May 2016 in the university hospital “Federico II” of Naples. The Amsterdam Preoperative Anxiety and Information Scale (APAIS) and the State-Trait Anxiety Inventory (STAI) were used to evaluate preoperative anxiety. In particular, APAIS measures preoperative anxiety and the need for information, and STAI assesses state and trait anxiety through STAI-Y1 and STAI-Y2, respectively. We analyzed APAIS and STAI scores in our population stratified on the basis of age, gender, marital status, previous surgical experiences, and type of surgery, and we compared them to our historical cohort. Statistical analysis was performed through a *t*-test and ANOVA for parametric data, and the Mann–Whitney and Kruskal–Wallis tests for non-parametric data. Linear regression was used to investigate the correlation between demographic data and the scores of STAI and APAIS in both groups.

**Results:**

Our results showed that state and preoperative anxiety remained stable, whereas trait anxiety increased in all the subgroups analyzed.

**Discussion:**

Even if state anxiety is considered a variable characteristic of the emotional sphere and trait anxiety a stable element, our findings suggested that COVID-19 deeply influenced trait anxiety, thus altering the patients’ psychological foundations.

## Introduction

Anxiety is a common unpleasant sensation experienced by many patients undergoing surgical procedures all over the world (e.g., USA 20–50%, Europe 27–70%, India 31–46%, and Africa 47–70%). It is defined as a stress response to an unfocused threat, whereas fear is defined as the reaction to a specific danger (1–12). Anxiety can cause a state of exaggerated alertness, often in association with physical and psychological symptoms, such as restlessness, fatigue, muscular tension, sweating, tachycardia, increased blood pressure, intrusive thoughts, and difficulty maintaining concentration (8, 13, 14). Physical symptoms are triggered by the activation of the autonomic nervous system, which leads to the neuroendocrine response through an augmented release of many hormones, such as catecholamines, cortisol, and prolactin, thus increasing the risk of both intraoperative and postoperative complications, such as hemodynamic instability, pulmonary complications, and mental disorders, and higher consumption of anesthetic drugs (15–17). Some studies showed that gynecological elective surgical patients with a high anxiety trait need an increased dose of propofol for the induction and maintenance of anesthesia, while anxious patients undergoing cholecystectomy have a higher risk of intraoperative hemodynamic events (15, 16). Moreover, as previously reported, the correlation between preoperative anxiety and the reduction of pain threshold leads to an increased requirement for analgesic drugs (14, 18–22).

Preoperative anxiety can have a multifactorial origin as it can be influenced by gender, age, social status, and previous surgical experiences (23, 24).

Notably, anxiety can lead to an impairment of the immune system and, consequently, to a higher risk of infections (25).

Our study aimed to determine the level of preoperative anxiety during the COVID-19 pandemic; in addition, we compared it to the preoperative anxiety of a population screened in a previous study conducted in our center to investigate if there is any difference between pre-pandemic and pandemic times (26).

## Materials and methods

The present study was approved by the local ethic committee “Comitato Etico Università Federico II” (protocol number: 155/20). After explaining the purpose of our research and obtaining written informed consent, we enrolled 314 patients undergoing elective surgery between May 2021 and November 2021 at University Hospital “Federico II” of Naples; they were asked to fill out the questionnaires during the pre-surgical anesthesia assessment. The historical cohort was obtained from the database of a previous study that we conducted before the pandemic (from July 2015 to May 2016) on 122 patients to validate the Italian version of APAIS in the same hospital (27).

The inclusion criteria were as follows: patients in the age bracket of 18–75 years, with adequate language skills, and appropriate comprehension of the questionnaires.

The exclusion criteria were as follows: patients undergoing emergency surgery or obstetric procedures, patients enrolled in our previous study, patients with psychiatric diseases, and patients using psychotropic drugs.

We recorded the following data: anthropometric measures, marital status, educational level, family status, medical and clinical history, and previous surgery. We used two tests to investigate preoperative anxiety, the STAI questionnaire, which is divided into two sections each of them consisting of 20 questions (Y1 and Y2), and the APAIS, a 6-item questionnaire. STAI Y1 was used to measure state anxiety, whereas STAI-Y2 assessed trait anxiety of the patients. State anxiety is defined as a transitory emotional response, whereas trait anxiety is a stable psychological characteristic of the individual, describing the probability to feel anxious in a distressing situation (28). Furthermore, we used APAIS to evaluate not only the anxiety related to anesthesia and the surgery but also the need for information about them. The higher the scores of STAI and APAIS, the higher the level of anxiety of the patient.

## Statistical analysis

We first performed a Shapiro–Wilk test to assess the normal distribution of the data. Parametric data were presented as mean and standard deviation, non-parametric data as median and interquartile range, and categorical variables as frequencies. Parametric data were analyzed through Student’s *t*-test and the analysis of variance (ANOVA), non-parametric data were analyzed through the Mann–Whitney and Kruskal–Wallis tests, and Pearson’s chi-square test was used to compare frequencies. *Post hoc* analyses were performed with Tukey’s test for parametric data and Dunn’s test for non-parametric data. We conducted a multivariate analysis through a linear model to correlate STAI-Y1, STAI-Y2, and APAIS scores to the demographic data such as age, gender, marital status, previous surgery, and type of surgery. Differences were considered statistically significant if *p* < 0.05. All tests were performed using R Studio (28).

## Results

We collected data from 122 patients before the pandemic and 314 patients during the pandemic. Socio-demographic characteristics are reported in Table 1. The two groups differed in gender composition, marital status, comorbidity, previous surgery, American Society of Anesthesiologists (ASA) status, and type of surgery. Table 2 reports the analysis of STAI-Y1; there was no difference in the scores reported by the two groups except for the subgroups of patients aged 30–39 years old and men, which showed an increased level of state anxiety during the pandemic.

**TABLE 1:** Demographic characteristics of the study population.

	Pre-pandemic		During-pandemic		
			
	*N* (%)	Mean (SD)	*N* (%)	Mean (SD)	*P*-value
All	122 (100%)		314 (100%)		
Age (years)	122 (100%)	50.1 (14.26)	314 (100%)	48.05 (16.62)	0.072
18–29	14 (11%)	24.42 (3.39)	44 (14%)	23.79 (3.36)	0.54
30–39	11 (9%)	34.38 (3.73)	62 (20%)	34.64 (3.03)	0.78
40–49	29 (24%)	43.86 (2.91)	73 (23%)	44.87 (2.3)	0.06
50–59	26 (21%)	54.73 (2.77)	51 (16%)	55.19 (2.72)	0.48
>60	42 (32%)	65.45 (4.95)	84 (17%)	67.28 (4.96)	0.056
Gender					<0.001
Male	70 (57%)		93 (30%)		0.07
Female	52 (43%)		221 (70%)		<0.001
Height (cm)	168.05 (8.337)		164.82 (37.34)		0.261
BMI (kg/cm^2^)	168.05 (8.337)		164.82 (37.34)		0.190
Marital status					0.001
Married	98 (80%)		202 (64%)		<0.001
Not married	24 (20%)		112 (36%)		<0.001
Race					0.925
Caucasic	122 (100%)		312 (99%)		
Asian	0 (0%)		2 (1%)		
Comorbidity					<0.001
Yes	38 (31%)		236 (75%)		<0.001
No	84 (69%)		78 (25%)		0.63
Previous surgery					<0.001
Yes	111 (90%)		79 (25%)		0.02
No	11 (10%)		235 (75%)		<0.001
ASA					<0.001
I	20 (16%)		39 (12%)		0.001
II	73 (60%)		233 (74%)		<0.001
III	28 (23%)		38 (12%)		0.21
IV	1 (1%)		0 (0%)		0.31
Type of surgery					<0.001
Minor	20 (17%)		44 (14%)		1
Intermediate	59 (48%)		53 (18%)		<0.001
Major	43 (35%)		217 (68%)		<0.001

**TABLE 2:** STAY-Y1 score for pre-pandemic and during-pandemic groups.

	Pre-pandemic		During pandemic		*P*-value
			
	*N* (%)	Median (Q1–Q3)	*N* (%)	Median (Q1–Q3)	
All	122 (100%)	43 (35–51)	314 (100%)	45 (42–49)	0.05
**Age (years)**					
18–29	14 (11%)	45.5 (32.5–54.25)	44 (14%)	46 (42–48)	0.799
30–39	11 (9%)	36 (32–49)	62 (20%)	45 (42–49.5)	0.021
40–49	29 (24%)	42 (35.5–42)	73 (23%)	45.5 (42–49)	0.48
50–59	26 (21%)	45.5 (33.75–59.75)	51 (16%)	45 (40–49)	0.944
>60	42 (32%)	43.5 (37–50.75)	84 (17%)	45 (42.25–48)	0.189
*p*-value		0.809		0.954	
**Gender**					
Male	70 (57%)	32.5 (32–44)	93 (30%)	46 (43–49)	<0.001
Female	52 (43%)	47 (36.5–56)	221 (70%)	45 (41–49)	0.57
*p*-value		<0.001		0.151	
**Marital status**					
Married	98 (80%)	43.5 (36–51)	202 (64%)	45 (42–49)	0.058
Not married	24 (20%)	39 (33–51.25)	112 (36%)	44 (41–48)	0.262
*p*-value		0.459		0.033	
**Previous surgery**					
Yes	111 (90%)	43 (36–51)	79 (25%)	45 (41–48)	0.425
No	11 (10%)	38 (32–52)	235 (75%)	46 (42–49)	0.145
*p*-value		0.426		0.374	
**Type of surgery**					
Minor	20 (17%)	47.5 (35.5–47.5)	44 (14%)	44 (41.25–48.75)	0.728
Intermediate	59 (48%)	43 (37–52)	53 (18%)	46 (41–49)	0.413
Major	43 (35%)	39 (33–50)	217 (68%)	45 (42–49)	0.09
*p*-value		0.33		0.9	

Table 3 shows that STAI-Y2 scores were significantly higher in the during-pandemic subgroups than in pre-pandemic ones, except for the subgroups aged 50–59 years old and subjects undergoing minor surgery.

**TABLE 3:** STAY-Y2 score for pre-pandemic and during-pandemic groups.

	Pre-pandemic		During pandemic		*P*-value
			
	*N* (%)	Median (Q1–Q3)	*N* (%)	Median (Q1–Q3)	
All	122 (100%)	37.5 (30.75–44)	314 (100%)	46 (42–49)	<0.001
**Age (years)**					
18–29	14 (11%)	35.5 (29.5–42.75)	44 (14%)	47 (43–49)	<0.001
30–39	11 (9%)	33 (30–42)	62 (20%)	45.5 (41–48.25)	<0.001
40–49	29 (24%)	36 (31.5–42)	73 (23%)	44 (41–48)	<0.001
50–59	26 (21%)	40.5 (34.25–49.25)	51 (16%)	45 (41–50)	0.031
>60	42 (32%)	38 (29.25–42.75)	84 (17%)	46 (43–49.75)	<0.001
*p*-value		0.482		0.135	
**Gender**					
Male	70 (57%)	36 (29–42)	93 (30%)	46 (42–48.5)	<0.001
Female	52 (43%)	40 (33–45)	221 (70%)	46 (41–49)	<0.001
*p*-value		0.08		0.679	
**Marital status**					
Married	98 (80%)	38 (32–44)	202 (64%)	45 (42–49)	<0.001
Not married	24 (20%)	35.5 (30–42)	112 (36%)	46 (41–49)	<0.001
*p*-value		0.308		0.78	
**Previous surgery**					
Yes	111 (90%)	38 (32–44)	79 (25%)	44 (41–48)	<0.001
No	11 (10%)	32 (29–35)	235 (75%)	46 (43–49)	<0.001
*p*-value		0.083		0.081	
**Type of surgery**					
Minor	20 (17%)	42.5 (36.75–42.5)	44 (14%)	46.5 (41–48)	0.094
Intermediate	59 (48%)	38 (30-44)*	57 (18%)	45 (42.5–48.5)	<0.001
Major	43 (35%)	34 (29–41)^†^	217 (68%)	46 (41.5–49)	<0.001
*p*-value		0.005		0.9	

*There is a statistically significant difference between intermediate and minor surgery (*p* = 0.046).

^†^There’s a statistically significant difference between major and minor surgery (*p* < 0.001).

Table 4 shows the overall APAIS score. No significant differences were detected between pre- and during-pandemic groups, except for men and patients undergoing minor surgery, which showed a lower score during the pandemic.

**TABLE 4:** The Amsterdam Preoperative Anxiety and Information Scale (APAIS) score for pre-pandemic and during-pandemic groups.

	Pre-pandemic		During pandemic		*P*-value
			
	*N* (%)	Median (Q1–Q3)	*N* (%)	Median (Q1–Q3)	
All	122 (100%)	15 (10–21)	318 (100%)	15 (11–21)	0.834
**Age (years)**					
18–29	14 (11%)	17.5 (11.75–20.5)	44 (14%)	15 (7.25–20)	0.16
30–39	11 (9%)	14 (11–21)	62 (20%)	16.5 (12–23.25)	0.521
40–49	29 (24%)	16 (12.5–18)	73 (23%)	15 (10–20.75)	0.941
50–59	26 (21%)	17 (10.75–22)	51 (16%)	14 (10–20)	0.493
>60	42 (32%)	14.5 (10–21.75)	84 (17%)	16 (12–20.75)	0.748
*p*-value		0.881		0.339	
**Gender**					
Male	70 (57%)	14 (10–18)	93 (30%)	12 (6–12)	0.011
Female	52 (43%)	17 (12.5–22)	221 (70%)	17 (12–22)	0.893
*p*-value		0.029		<0.001	
**Marital status**					
Married	98 (80%)	15 (11–21)	202 (64%)	15 (10–21)	0.649
Not married	24 (20%)	14.5 (10.25–19.5)	112 (36%)	15 (10–21)	0.786
*p*-value		0.633		0.768	
**Previous surgery**					
Yes	111 (90%)	15 (11–21)	79 (25%)	15 (9–21)	0.911
No	11 (10%)	15 (11–20)	235 (75%)	15 (10–21)	0.854
*p*-value		0.778		0.988	
**Type of surgery**					
Minor	20 (17%)	17 (10–22)	44 (14%)	12 (6–16)	0.02
Intermediate	59 (48%)	16 (11–21)	57 (18%)	15 (10–20.5)*	0.51
Major	43 (35%)	14 (11–20)	217 (68%)	17 (12–21)^†^	0.168
*p*-value		0.695		<0.001	

*There is a statistically significant difference between intermediate and minor surgery (*p* = 0.046).

^†^There is a statistically significant difference between major and minor surgery (*p* < 0.001).

Supplementary Table 1 reports the score obtained from the sum of APAIS questions about the anxiety correlated to anesthesia and surgery; men experienced a lower level of anxiety during the pandemic compared to the pre-pandemic group. In both groups, it is confirmed that women are more anxious than men about anesthesia and surgery.

Supplementary Table 2 shows the score obtained from the sum of APAIS questions about the need for information about anesthesia and surgery. No significant differences were recorded between pre- and during-pandemic groups.

Multivariate analysis showed that during the pandemic, STAI-Y1 is inversely correlated to age between 30 and 39 [standardized beta coefficient −2.71, 95% confidence interval (CI): (−5.34; −0.08), *p*-value: 0.043], while no correlation was found between STAI-Y1 score and the other demographic data analyzed both during and before the pandemic (Supplementary Table 3). STAI-Y2 score inversely correlated only to the male gender before the pandemic (standardized beta coefficient: −3.44; 95% CI: −6.60; −0.29, *p*-value: 0.033; Supplementary Table 4). APAIS score inversely correlated to the male gender both before the pandemic (standardized beta coefficient: −2.54, 95% CI: −4.70; −0.37, *p*-value: 0.022) and during the pandemic (standardized beta coefficient: −3.94, 95% CI: −5.62; −2.25, *p*-value: < 0.001) and inversely correlated to age more than 60 during the pandemic (standardized beta coefficient: 3.16, 95% CI: −5.69; −0.62, *p*-value: 0.015; Supplementary Table 5).

## Discussion

Our study aimed to investigate the difference in preoperative anxiety before and during the pandemic. We found that according to STAI-Y1, there were no significant changes in state anxiety; this result was confirmed by APAIS, which did not show any modification during the pandemic for both the anxiety and the need for information subscales. Notably, all the subgroups analyzed showed increased trait anxiety during the pandemic, as recorded by STAI-Y2.

A possible explanation of this result might be the measures adopted to face the spread of the infection in the hospital; in fact, there was close monitoring of patients before admission to the hospital, checking for the virus infection through nasopharyngeal swabs. Apparently, this initial screening probably made the patient feel safe from the contagion.

Previous studies confirmed our result that age does not significantly affect preoperative anxiety, even during the COVID-19 pandemic; only older patients showed a significantly higher level of perioperative anxiety measured by APAIS during the pandemic, maybe because they felt to be a more fragile category of patients based on information conveyed by mass media (23). According to STAI-Y1, only young adults seemed to experience a higher level of anxiety during the pandemic compared to the pre-pandemic period, probably due to their greater and easier access to the internet and social media, which may have increased the exposure to misinformation; furthermore, men showed an increased level of state anxiety during the pandemic (29). This finding is in contrast with the APAIS score, which showed that during the pandemic preoperative anxiety about anesthesia and surgery in men was lower than in the pre-pandemic period; it is important to underline that APAIS questions are specifically focused on anesthesia and surgery, while STAI-Y1 questions are more generic and the patient could forget that the questionnaire refers to the procedures they will undergo (30). In addition, our data confirmed that both during and before the pandemic, the level of preoperative anxiety was lower in men than in women (23).

In accordance with other studies, previous surgical experiences, type of surgery, and marital status did not influence preoperative anxiety both during and before the pandemic (23, 31, 32).

Notably, while overall anxiety did not seem to be substantially affected by the pandemic, trait anxiety augmented in all the subgroups. These findings agree with previous studies investigating trait anxiety in different populations during the pandemic. Trait anxiety is a relatively stable characteristic of a patient’s psychology, but it can be modified by many factors such as psychological therapies; we found that the psychological burden of the pandemic was so powerful to be able to alter the hardcore of people’s emotional sphere (30, 33). The COVID-19 pandemic did impact the socio-economic, relational, and working aspects as well, and all these factors can profoundly influence the patient’s mood (34–38).

Our study has some limitations. First, pre-pandemic and during-pandemic groups are not homogenous in gender, comorbidity, previous surgery, and type of surgery. Notably, all the above-mentioned differences recorded in our groups were supposed to lead to a higher level of state anxiety during the pandemic, but we found that state anxiety was not significantly different between before and during the pandemic. Second, we investigated preoperative anxiety in our hospital; consequently, our findings must be cautiously interpreted and cannot be generalized.

In conclusion, preoperative anxiety is a fundamental issue for many patients undergoing elective surgery, and it could influence perioperative management. The COVID-19 pandemic had an important impact on the psychology of people. Our work highlighted that even if the anxiety related to surgery and anesthesia was not significantly modified during the pandemic, the COVID-19 outbreak has been able to deeply alter the emotional sphere of the patient, revealing that trait anxiety is not an unchangeable characteristic of the subject, but it can be modeled by important socio-economic changes.

## Data availability statement

The raw data supporting the conclusions of this article will be made available by the authors, without undue reservation.

## Ethics statement

The studies involving human participants were reviewed and approved by “Comitato Etico Università Federico II”. The patients/participants provided their written informed consent to participate in this study.

## Author contributions

PB conceived the study and supervised the work. AM wrote the manuscript. MV conducted statistical analyses. CI helped to supervise the project. SN collected and organized the data. AS analyzed the data and drafted the manuscript. GS critically revised the manuscript. All authors contributed to the article and approved the submitted version.

## Supplementary material

Supplementary Table 1 APAIS anxiety score for pre-pandemic and during-pandemic groups.

Supplementary Table 2 APAIS need for information score for pre-pandemic and during-pandemic groups.

Supplementary Table 3 Standardized beta coefficients of multivariate analysis analyzing the relationship between during and pre-pandemic STAI-Y1 score and demographic data.

Supplementary Table 4 Standardized beta coefficients of multivariate analysis analyzing the relationship between during and pre-pandemic STAI-Y2 score and demographic data.

Supplementary Table 5 Standardized beta coefficients of multivariate analysis analyzing the relationship between during and pre-pandemic APAIS score and demographic data.
