# Supplementary material for: Preoperative anxiety during COVID-19 pandemic: A single-center observational study and comparison with a historical cohort
Source: Front Med (Lausanne). 2022 Dec 15;9:1062381. doi: 10.3389/fmed.2022.1062381 (PMC9797972; doi:10.3389/fmed.2022.1062381)
Supplement: Supplementary Table 1 — APAIS anxiety score for pre-pandemic and during-pandemic groups. [file Table_1.docx]

**Table S1**. APAIS anxiety score for pre-pandemic and during pandemic groups

|  | **Pre-Pandemic** | | |  | **During Pandemic** | |  | **p-value** |
| --- | --- | --- | --- | --- | --- | --- | --- | --- |
|  | N (%) | Median (Q1-Q3) |  | | N (%) | Median (Q1-Q3) |  |  |
| All | 122 (100%) | 10 (7-14) | |  | 318 (100%) | 10 (6-14) |  | 0,632 |
| Age (y) |  |  | |  |  |  |  |  |
| 18-29 | 14 (11%) | 9,5 (7,5-14,25) | |  | 44 (14%) | 10 (8-13) |  | 0,942 |
| 30-39 | 11 (9%) | 9 (7-11) | |  | 62 (20%) | 10 (7-14) |  | 0,435 |
| 40-49 | 29 (24%) | 10 (8-12,5) | |  | 73 (23%) | 10 (7-14) |  | 0,988 |
| 50-59 | 26 (21%) | 9,5 (5,75-15,25) | |  | 51 (16%) | 12 (7-15) |  | 0,705 |
| >60 | 42 (32%) | 10 (5,75-14) | |  | 84 (17%) | 8 (4-12)* |  | 0,062 |
| p-value |  | 0,99 | |  |  | 0,013 |  |  |
| Gender | | | | | | | | |
| Male | 70 (57%) | 9,5 (5,75-12,25) | |  | 93 (30%) | 7 (4-11) |  | 0,003 |
| Female | 52 (43%) | 10 (8-5) | |  | 221 (70%) | 11 (8-14) |  | 0,771 |
| p-value |  | 0,024 | |  |  | <0,001 |  |  |
| Marital Status | | | | | | | | |
| Married | 98 (80%) | 10 (7-14) | |  | 202 (64%) | 10 (7-14) |  | 0,555 |
| Not married | 24 (20%) | 9 (6,25-11,75) | |  | 112 (36%) | 10 (6-13,75) |  | 0,644 |
| p-value |  | 0,255 | |  |  | 0,575 |  |  |
| previous Surgery | | | | | | | | |
| Yes | 111 (90%) | 10 (7-13) | |  | 79 (25%) | 10 (6-15) |  | 0,955 |
| No | 11 (10%) | 8 (6-14) | |  | 235 (75%) | 10 (6-13) |  | 0,99 |
| p-value |  | 0,750 | |  |  | 0,533 |  |  |
| Type of surgery | | | | | | | | |
| Minor | 20 (17%) | 7,5 (5-13,75) | |  | 44 (14%) | 6 (4-11,75) |  | 0,251 |
| Intermediate | 59 (48%) | 10 (8-13) | |  | 57 (18%) | 9 (6-13) |  | 0,47 |
| Major | 43 (35%) | 11 (8-15) | |  | 217 (68%) | 11 (7-14) ^†^ |  | 0,663 |
| p-value |  | 0,174 | |  |  | <0,001 |  |  |

*there is a statistical significant difference between patients aged 50-59 and patients aged >60 (p=0,032); ^†^ there is a statistical significant difference between minor and major surgery (p<0,001).
