# Supplementary material for: Preoperative anxiety during COVID-19 pandemic: A single-center observational study and comparison with a historical cohort
Source: Front Med (Lausanne). 2022 Dec 15;9:1062381. doi: 10.3389/fmed.2022.1062381 (PMC9797972; doi:10.3389/fmed.2022.1062381)
Supplement: Supplementary Table 2 — APAIS need for information score for pre-pandemic and during-pandemic groups. [file Table_2.docx]

**Table S2**. APAIS need for information score for pre-pandemic and during pandemic groups

|  | **Pre-Pandemic** | | |  | **During Pandemic** | |  | **p-value** |
| --- | --- | --- | --- | --- | --- | --- | --- | --- |
|  | N (%) | Median (Q1-Q3) |  | | N (%) | Median (Q1-Q3) |  |  |
| All | 122 (100%) | 5 (3-7,25) | |  | 318 (100%) | 6 (3-8) |  | 0,604 |
| Age (y) |  |  | |  |  |  |  |  |
| 18-29 | 14 (11%) | 6 (4,75-8,25) | |  | 44 (14%) | 6 (5-8) |  | 0,978 |
| 30-39 | 11 (9%) | 5 (4-6) | |  | 62 (20%) | 6 (3,75-9) |  | 0,237 |
| 40-49 | 29 (24%) | 5 (2,5-6,5) | |  | 73 (23%) | 6 (3-8) |  | 0,139 |
| 50-59 | 26 (21%) | 6 (3,75-8) | |  | 51 (16%) | 5 (3-7) |  | 0,453 |
| >60 | 42 (32%) | 4,5 (3-8) | |  | 84 (17%) | 4,5 (2-6,75) * |  | 0,189 |
| p-value |  | 0,340 | |  |  | 0,003 |  |  |
| Gender | | | | | | | | |
| Male | 70 (57%) | 5,5 (3-7) | |  | 93 (30%) | 4 (2-6) |  | 0,068 |
| Female | 52 (43%) | 5 (4-8) | |  | 221 (70%) | 6 (4-8) |  | 0,272 |
| p-value |  | 0,707 | |  |  | <0,001 |  |  |
| Marital Status | | | | | | | | |
| Married | 98 (80%) | 5,5 (3-7,25) | |  | 202 (64%) | 5 (3-8) |  | 0,667 |
| Not married | 24 (20%) | 5 (4-7,75) | |  | 112 (36%) | 6 (3-8) |  | 0,902 |
| p-value |  | 0,349 | |  |  | 0,4 |  |  |
| previous Surgery | | | | | | | | |
| Yes | 111 (90%) | 5 (3-8) | |  | 79 (25%) | 6 (3-8) |  | 0,745 |
| No | 11 (10%) | 5 (4-7) | |  | 235 (75%) | 6 (3-8) |  | 0,799 |
| p-value |  | 0,924 | |  |  | 0,999 |  |  |
| Type of surgery | | | | | | | | |
| Minor | 20 (17%) | 4 (2-6) | |  | 44 (14%) | 4 (2-6) |  | 0,772 |
| Intermediate | 59 (48%) | 6 (4-8) | |  | 57 (18%) | 6 (3-8) |  | 0,785 |
| Major | 43 (35%) | 6 (3-7) | |  | 217 (68%) | 6 (3,5-8) ^†^ |  | 0,318 |
| p-value |  | 0,159 | |  |  | 0,007 |  |  |

*there is a statistical significant difference between patients aged >60 and patients aged 30-39 (p=0.023) and between patients aged >60 and patients aged 18-29 (p=0.008); ^†^ there is a statistical significant difference between major and minor surgery (p<0,001) and between major and intermediate surgery (p=0.025).
