# Supplementary material for: Preoperative anxiety during COVID-19 pandemic: A single-center observational study and comparison with a historical cohort
Source: Front Med (Lausanne). 2022 Dec 15;9:1062381. doi: 10.3389/fmed.2022.1062381 (PMC9797972; doi:10.3389/fmed.2022.1062381)
Supplement: Supplementary Table 3 — Standardized beta coefficients of multivariate analysis analyzing the relationship between during and pre-pandemic STAI-Y1 score and demographic data. [file Table_3.docx]

**Table S3.** Standardized beta coefficients of multivariate analysis analyzing the relationship between during and pre-pandemic STAI-Y1 score and demographic data.

|  | **STAI-Y1** | | | |
| --- | --- | --- | --- | --- |
|  | **Pre-Pandemic** | | **During Pandemic** | |
|  | Standardized beta coefficient (CI 95%) | p value | Standardized beta coefficient (CI 95%) | p value |
| Age (y) |  |  |  |  |
| 18-29 | ref |  | ref |  |
| 30-39 | -5.24 (-15.24; 4.75) | 0.301 | -2.71 (-5.34; -0.08) | **0.043** |
| 40-49 | -1.52 (-10.92; 7.88) | 0.749 | -1.44 (-4.04; 1.17) | 0.279 |
| 50-59 | -1.21(-10.80; 8.38) | 0.803 | 0.31(-2.47; 3.10) | 0.825 |
| >60 | -3.25 (-12.55; 6.06) | 0.491 | -2.07 (-4.70; 0.55) | 0.121 |
|  |  |  |  |  |
| Gender | | | | |
| Female | ref |  | ref |  |
| Male | -3.47 (-7.49; 0.56) | 0.091 | 0.74 (-1.01; 2.48) | 0.406 |
|  |  |  |  |  |
| Marital Status | | | | |
| Married | ref |  | ref |  |
| Not married | -1.53 (-8.30; 5.24) | 0.655 | -1.37 (-3.01; 0.28) | 0.103 |
|  |  |  |  |  |
| Previous surgery | | | | |
| No | ref |  | ref |  |
| Yes | 1.53 (-5.99; 9.05) | 0.688 | -0.96 (-2.68; 0.76) | 0.271 |
|  |  |  |  |  |
| Type of surgery | | | | |
| Minor | 1.09 (-4.84; 7.03) | 0.716 | -0.29 (-3.13; 2.56) | 0.842 |
| Intermediate | ref |  | ref |  |
| Major | 3.95 (-0.45; 8.35) | 0.078 | -0.14 (-2.21; 1.94) | 0.897 |
