# Supplementary material for: Preoperative anxiety during COVID-19 pandemic: A single-center observational study and comparison with a historical cohort
Source: Front Med (Lausanne). 2022 Dec 15;9:1062381. doi: 10.3389/fmed.2022.1062381 (PMC9797972; doi:10.3389/fmed.2022.1062381)
Supplement: Supplementary Table 4 — Standardized beta coefficients of multivariate analysis analyzing the relationship between during and pre-pandemic STAI-Y2 score and demographic data. [file Table_4.docx]

Table S4. Standardized beta coefficients of multivariate analysis analyzing the relationship between during and pre-pandemic STAI-Y2 score and demographic data

|  | **STAI-Y2** | | | |
| --- | --- | --- | --- | --- |
|  | **Pre-Pandemic** | | **During Pandemic** | |
|  | Standardized beta coefficient (CI 95%) | p value | Standardized beta coefficient (CI 95%) | p value |
| Age (y) |  |  |  |  |
| 18-29 | ref |  | ref |  |
| 30-39 | -4.31 (-12.13; 3.52) | 0.278 | -0.25 (-2.56; 2.07) | 0.833 |
| 40-49 | -3.58 (-10.94; 3.78) | 0.337 | -1.34 (-3.64; 0.95) | 0.250 |
| 50-59 | 0.74 (-6.77; 8.24) | 0.846 | -0.09 (-2.55; 2.36) | 0.942 |
| >60 | -3.44 (-10.72; 3.84) | 0.351 | -1.35 (-3.66; 0.96) | 0.251 |
|  |  |  |  |  |
| Gender | | | | |
| Female | ref |  | ref |  |
| Male | -3.44 (-6.60; -0.29) | **0.033** | 0.61 (-0.93; 2.15) | 0.434 |
|  |  |  |  |  |
| Marital Status | | | | |
| Married | ref |  | ref |  |
| Not married | -2.33 (-7.63; 2.96) | 0.384 | 0.08 (-1.37; 1.53) | 0.915 |
|  |  |  |  |  |
| Previous surgery | | | | |
| No | ref |  | ref |  |
| Yes | 5.72 (-0.17; 11.60) | 0.057 | -1.02 (-2.53; 0.49) | 0.186 |
|  |  |  |  |  |
| Type of surgery | | | | |
| Minor | -1.78 (-6.43; 2.86) | 0.449 | -0.52 (-3.03; 1.98) | 0.682 |
| Intermediate | ref |  | ref |  |
| Major | -0.29 (-3.73 – 3.15) | 0.869 | 0.07 (-1.76; 1.90) | 0.941 |
