# Supplementary material for: Preoperative anxiety during COVID-19 pandemic: A single-center observational study and comparison with a historical cohort
Source: Front Med (Lausanne). 2022 Dec 15;9:1062381. doi: 10.3389/fmed.2022.1062381 (PMC9797972; doi:10.3389/fmed.2022.1062381)
Supplement: Supplementary Table 5 — Standardized beta coefficients of multivariate analysis analyzing the relationship between during and pre-pandemic APAIS score and demographic data. [file Table_5.docx]

Table S5. Standardized beta coefficients of multivariate analysis analyzing the relationship between during and pre-pandemic APAIS score and demographic data.

|  | **APAIS** | | | |
| --- | --- | --- | --- | --- |
|  | **Pre-Pandemic** | | **During Pandemic** | |
|  | Standardized beta coefficient (CI 95%) | p value | Standardized beta coefficient (CI 95%) | p value |
| Age (y) |  |  |  |  |
| 18-29 | ref |  | ref |  |
| 30-39 | -3.01 (-8.39; 2.36) | 0.269 | -1.06 (-3.59; 1.48) | 0.414 |
| 40-49 | -4.25 (-9.30; 0.80) | 0.098 | -2.13 (-4.64; 0.39) | 0.097 |
| 50-59 | -1.78 (-6.94; 3.37) | 0.495 | -1.48 (-4.18; 1.21) | 0.280 |
| >60 | -2.66 (-7.66; 2.33) | 0.293 | -3.16 (-5.69; -0.62) | **0.015** |
|  |  |  |  |  |
| Gender | | | | |
| Female | ref |  | ref |  |
| Male | -2.54 (-4.70; -0.37) | **0.022** | -3.94 (-5.62; -2.25) | **<0.001** |
|  |  |  |  |  |
| Marital Status | | | | |
| Married | ref |  | ref |  |
| Not married | -2.65 (-6.28, 0.99) | 0.152 | -0.74 (-2.32; 0.85) | 0.363 |
|  |  |  |  |  |
| Previous surgery | | | | |
| No | ref |  | ref |  |
| Yes | -1.02 (-5.06; 3.03) | 0.620 | 0.89 (-0.77; 2.56) | 0.291 |
|  |  |  |  |  |
| Type of surgery | | | | |
| Minor | -2.66 (-5.85; 0.53) | 0.102 | -1.27 (-4.02; 1.49) | 0.366 |
| Intermediate | ref |  | ref |  |
| Major | 0.11 (-2.26; 2.47) | 0.928 | 0.69 (-1.32; 2.70) | 0.498 |
